# World’s Oldest Case of Synchronous Bilateral Benign Phyllodes Tumors of the Breast: A Rare Occurrence

**DOI:** 10.7759/cureus.12281

**Published:** 2020-12-25

**Authors:** Shariful Islam, Anthony Maughn, Vinoo Bheem, Patrick Harnarayan, Vijay Naraynsingh

**Affiliations:** 1 General Surgery, San Fernando General Hospital, San Fernando, TTO; 2 Clinical Surgical Science, The University of the West Indies, St. Augustine, TTO; 3 Clinical Surgical Sciences, The University of the West Indies, St. Augustine, TTO; 4 Surgery, Medical Associates Hospital, St. Joseph, TTO

**Keywords:** phyllodes tumor, synchronous bilateral benign phyllodes, metachronous benign phyllodes tumor of the breast

## Abstract

Phyllodes tumors are rare fibroepithelial tumors of the breast. They account for less than 0.5% of breast cancers. Bilateral presentation is a rare event, and seems to be associated with the more benign subtype but, reports are scarce. It is more common to have multiple ipsilateral tumors or bilateral asynchronous presentations. However, bilateral synchronous phyllodes are seldom reported. A literature search has revealed only five cases of synchronous and one case of metachronous bilateral phyllodes tumor of the breast. The age ranges of these patients are between 16-42 years. We are reporting the world’s first case of bilateral synchronous phyllodes tumor of the breast in a patient over the age of 50 years.

## Introduction

Phyllodes tumors are rare fibroepithelial tumors of the breast which range in their spectrum of behavior from benign to overtly malignant. It was first coined as “cystosarcoma Phyllodes” by John Muler in 1838 to describe its cystic and fleshy appearance and not its malignant potential. Several terms have been used to describe this entity until the adoption of the name Phyllodes tumor by the World Health Organization. They account for less than 0.5% of breast cancers with a median age of presentation of 42 to 45 years, being more common in Latino, Caucasian, and Asian populations [1]. Bilateral presentation is a rare event and seems to be associated with the more benign subtype but reports are scarce. Bilateral asynchronous or multiple ipsilateral presentations are more common. Here we are reporting the oldest patient in the literature, who presented with bilateral synchronous phyllodes tumors of the breast.

## Case presentation

**Figure 1:**
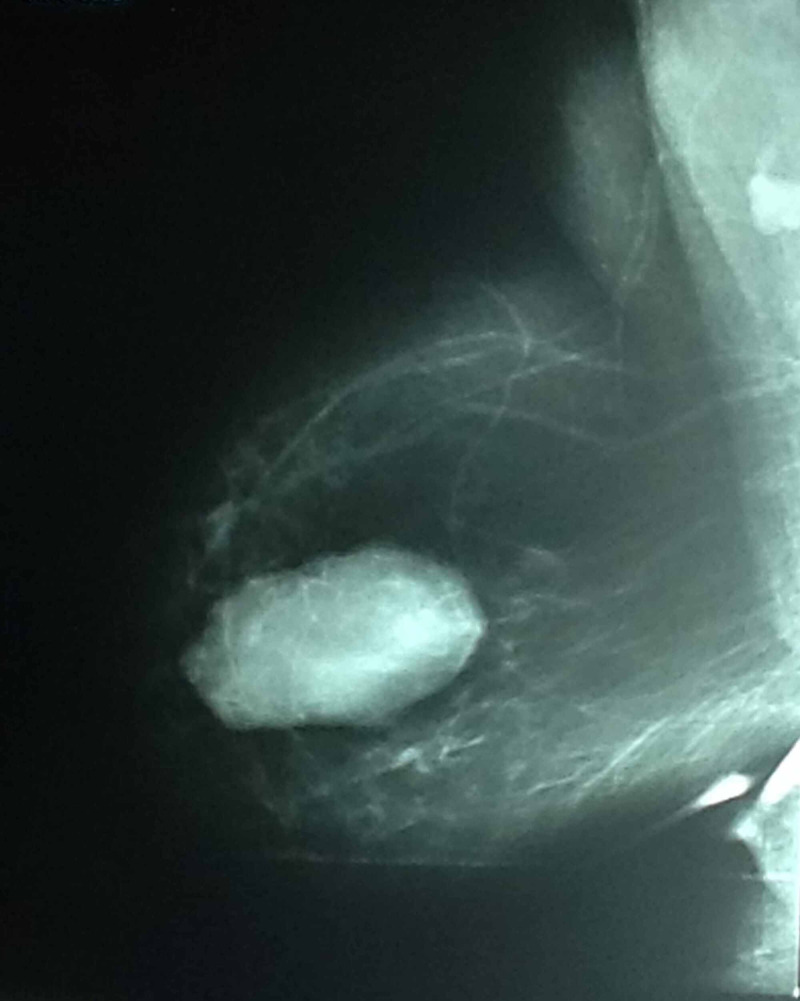
Mammogram of right breast showing an 8.2 cm x 5.3 cm well-defined lesion seen to the inner quadrant of the right breast

**Figure 2:**
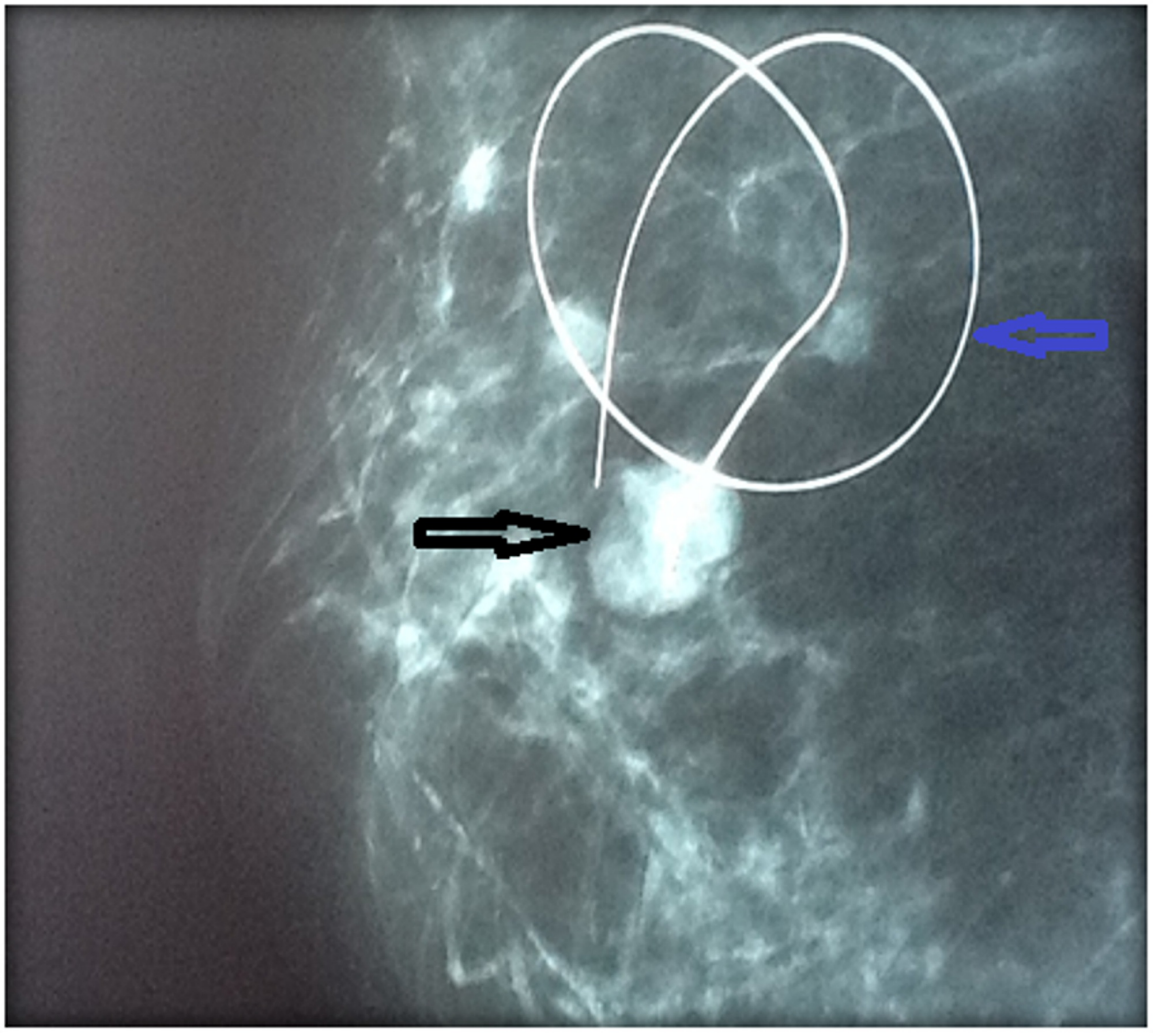
Mammogram of left breast showing a 2.5 cm x 1.5 cm lobulated lesion (black arrow) in the lower quadrant with wire localization (blue arrow)

This is the case of a 58-year-old female who is a known diabetic and hypertensive presented with a 9-month history of a pea-sized right breast lump. The lump was painful and it had recently increased in size. She was menarche at 15 years, had five children, and all of them were breastfed. The patient had a hysterectomy 20 years ago for fibroids and she never used oral contraception or hormone replacement therapy. She had no family history of breast or ovarian cancers and was otherwise fit and active. On examination, she was found to have a 6cm x 4cm lump in the upper inner quadrant of her right breast at the 2 o’clock position which was firm and mobile with no palpable lymphadenopathy. There was no obvious nipple discharge or skin changes. Her left breast examination was normal. Ultrasounds of her breast showed a 3.6cm x 4.2cm solid heterogeneous hyperechoic mass between 2 and 4 o’clock position in her right breast with no focal or solid lesion seen in the left breast. Bilateral mammograms showed scattered fibroglandularity to both breasts, with an 8.2cm x 5.3 cm well-defined lesion seen in the inner quadrant of her right breast (Figure 1) and a 2.5 cm x 1.5 cm lobulated lesion in the left in the lower quadrant (Figure 2). The report also revealed that there were bilateral axillary nodes with no adverse features. Core needle biopsies of the right breast lump and stereotactic biopsy of the left breast lump were performed and the histopathological result revealed bilateral benign phyllodes tumor of the breast.

**Figure 3:**
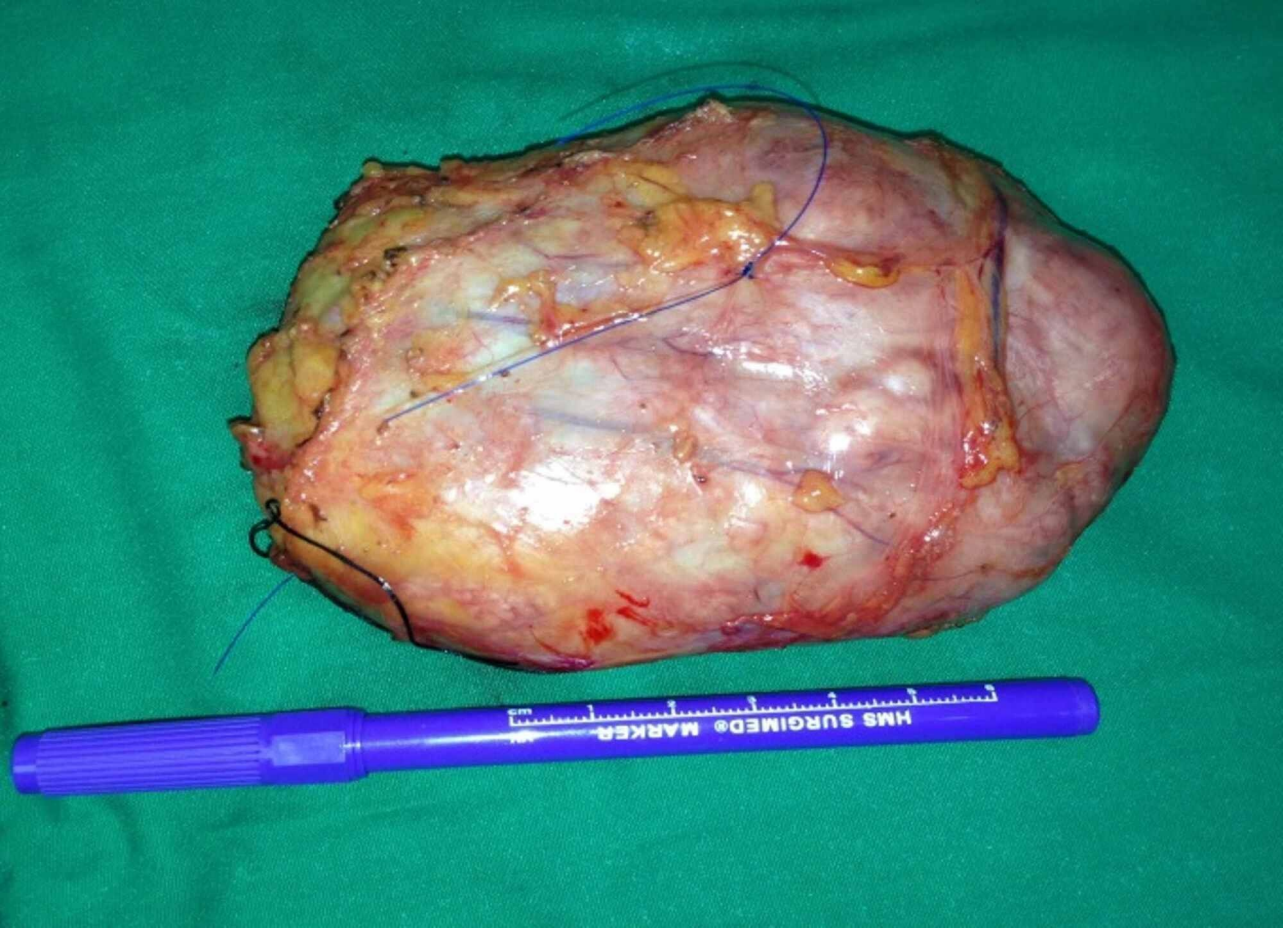
Intra op photograph of right phyllodes tumor showing the length of the tumor

**Figure 4:**
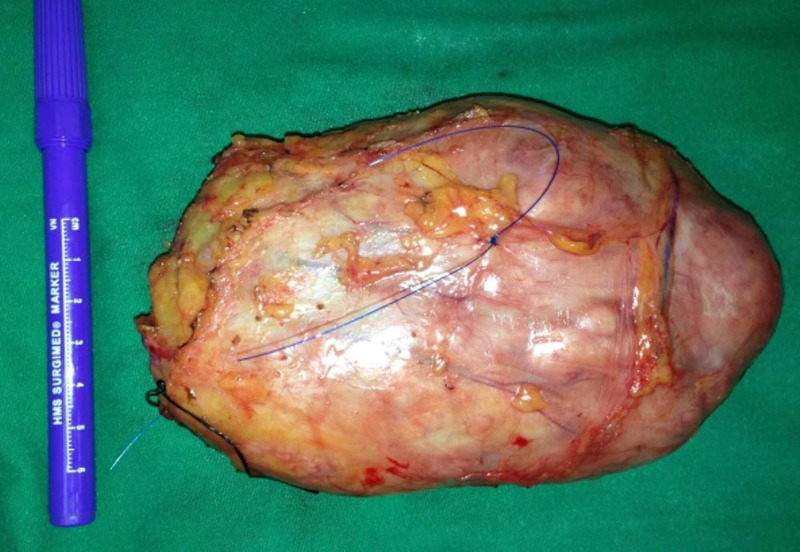
Intra op photograph of right phyllodes tumor showing the width of the tumor

The patient consented to bilateral wide local excision of the breast lumps. Preoperatively a guidewire was placed under stereotactic guidance in her left breast to localize the lump (Figure 2). The patient had wide local excision of her right breast lump (Figures 3, 4) and a wire-guided wide local excision of her left breast lump. Histology from these lesions showed typical features of a benign Phyllodes tumor with areas of atypical ductal hyperplasia and columnar cytomorphology. There was also marked stromal cellularity with mitotic activity below 2 per ten high-power fields. The resection margins were all negative and the closest margin was 1.5 cm away from the tumor.

The findings were discussed at the oncology multidiscipline meeting and started on adjuvant radiotherapy following surgery. She was followed up in a surgical outpatient clinic and noted to have no clinical or radiological recurrence at her three years of follow-up.

## Discussion

Phyllodes tumors are fibroepithelial tumors made up of mixed components of the epithelial and stromal elements which range from benign to malignant. They are more common in women but have been reported in men with gynecomastia [2]. No known aetiological or risk factors other than Li-Fraumeni syndrome (which presents with multiple tumors due to abnormalities of the p53 tumor suppressor gene) have been clearly identified. Grossly, they appear as multinodular greyish-white masses and microscopically they have typical leaf-like papillary projections of epithelial stroma with varying degrees of hyperplasia and atypia. Hence, the name “Phyllodes” means leaf-like.

Bilateral Phyllodes tumors are exceedingly rare. In a review of the clinicopathological features of Phyllodes tumors by Yoshidaya et al. in 2013, 91 patients diagnosed with Phyllodes tumor, only nine patients had multiple tumors (8.6%) and of these, eight were ipsilateral and one was bilateral [3]. Most reported cases are asynchronous with the largest number of synchronous tumors being 13 found in a 16-year-old female [1,4,5]. Recently Mallory et al. in 2015 reported excision of 15 (10 right, 5 left) bilateral phyllodes breast tumors in a 26-year-old nulliparous female [6].

A literature search has revealed only five cases of synchronous and one case of metachronous bilateral benign phyllodes tumor of the breast. The median age for phyllodes tumor varies in the literature [3], however, the median age for bilateral synchronous bilateral phyllodes is 22.8 years and it ranges between 16-42 years [3, 5-8]. Ezeome et al. in 2007 reported the only case of a metachronous bilateral benign phyllodes tumor in a 24-year-old nulliparous female patient [9]. The index case is a 58-year-old female with a bilateral synchronous presentation of benign phyllodes tumor. Literature has also reported the synchronous presentation of malignant and benign phyllodes tumors in one patient [10].

Phyllodes tumors rarely occur in pregnancy and lactation, as stromal hyperplasia is not a prominent feature during this period. However, this association is reported and the exact risk factors for this are unknown [2]. Three out of the five reported cases of synchronous bilateral phyllodes patients are nulliparous, one multiparous and in the last one, the parity was mentioned in the text [3, 5-8]. Our index case was multiparous and all the children were breastfed.

Clinically Phyllodes tumors are hard to differentiate from fibroadenomas. They usually present as firm mobile masses without skin or nipple involvement. However, 20% of these patients also present as a nonpalpable lump, detected only on screening investigations [11]. Malignant tumors rarely metastasize to the axilla and only seen in 20% of these patients. Pain is also atypical in their presentation. The size of the lumps is varied from a small lesion to a massive lesion distorting the breast. Any large or rapidly growing lesion and previously known benign lesion which has started to change should be suspected as phyllodes tumors. Radiologically, there are a few features to distinguish phyllodes tumors from the fibroadenomas other than size both mammographically and by ultrasound. Cystic areas within the lesion on ultrasound should draw your suspicion to Phyllodes tumors. MRI is not routinely used or recommended for diagnosis but may be useful in some patients in distinguishing Phyllodes tumors from fibroadenomas and carcinoma on T1-contrast enhance images [12]. It may also be useful in determining the extent of disease preoperatively and to guide appropriate treatment options.

In lesion suspected to be Phyllodes tumors, histological diagnosis is necessary either by biopsy or excision. Fine-needle aspiration cytology (FNAC) has a high false-negative rate while true-cut biopsies have a better sensitivity ranging from 70%-75% [13-15]. The use of immuno-histo-chemistry for the expression of Ki 67, p53, CD117, phospho-Histone3, mdm2, cdk4, and FBX4 S8R may improve the accuracy of diagnosis but have failed to predict outcomes.

Histology plays a central role in the determination of treatment of these tumors as the stromal elements or key in differentiating them from fibroadenomas and in distinguishing malignant from benign Phyllodes tumors. Histologically, they are classified into 1) benign, 2) borderline, and 3) malignant based on the a) mitotic activity, b) stromal overgrowth, c) pleomorphism and d) cellularity [14].

Histologically, benign tumors are characterized by having 1) <4 mitoses per 10 high power fields with mild stromal atypia, 2) increased stromal cellularity, 3) lack of stromal overgrowth and 4) having circumscribed tumor margins.

The borderline tumors have 1) more stromal cellularity and atypia, 2) lack stromal overgrowth, 3) have microscopic infiltrative borders, and 4) have between 4-9 mitosis per 10 high power fields.

Malignant tumors have 1) more than 10 mitoses, 2) have infiltrative borders, 3) marked stromal atypia, and 4) have an overgrowth of stromal elements.

The stromal elements of the phyllodes tumors are responsible for their malignant potential. These tumors behave differently than breast carcinomas, whose cellular elements predict their behavior. Therefore, the goal of treatment for these tumors is to achieve R (0) resections, either by wide local excision or mastectomy depending on the size of the tumor and the size of the breast. Axillary lymph node dissection is not routinely indicated as these tumors rarely metastasize to the axilla.

Wide local excision with 1 cm clear margins is recommended. For recurrent diseases or post-wide local cases where the margin less than 1 cm, a re-excision with adequate margins or mastectomy is often recommended.

Adjuvant radiotherapy, chemotherapy, and hormonal therapy have been investigated with the only radiotherapy showing some benefit in selected cases [16]. Radiotherapy has not been universally adopted in the treatment of Phyllodes tumors and has only been recommended by some for recurrence disease and in disease in which adequate margins cannot be gained [17]. Others have used it after local excision with positive margins or margins less than 1 cm. In these cases, re-excision with adequate margins or mastectomy is recommended. Chemotherapy and hormonal therapy have shown no benefit with respect to improving recurrence rate or overall disease survival and is not recommended [17].

Recurrence usually occurs within two years after adequate excision with a shorter time period for borderline and malignant disease. The main form of treatment for recurrence is re-excision or mastectomy to gain adequate margins followed by radiotherapy. Radiotherapy alone is indicated for recurrences in which excision is not possible to achieve local control, however, there is little data to suggest and guide optimal treatment for the recurrent disease [16].

Metastatic disease has been reported in 14% to 40% of patients with Phyllodes tumors with overall mean survival of 30 months. It usually presents in the lungs and if possible as with other soft tissue sarcoma resection is indicated. For unresectable disease, chemotherapy is used following the guidelines for soft tissue sarcomas with mixed results [17].

The prognosis of benign and borderline phyllodes tumors is good with adequate surgical excision. However, for the malignant tumors, the five-year survival rate ranges from 60%-80 [16]. There are no evidence-based guidelines for the surveillance of Phyllodes tumors and recommendations are based on those for soft tissue sarcomas. These patients should be followed up every six months for the first two years, then yearly with history, physical examination, and chest X-ray. However, in patients with high-risk features, the surveillance can be performed more often.

## Conclusions

Bilateral synchronous presentation of phyllodes tumor is a rare occurrence and seems to be associated with a benign subtype. It is reported in the younger patients. However, it can also occur in elderly females similar to our patient. It should be entertained in the differential diagnosis of all elderly patients presenting with a rapidly growing lump. The diagnosis can either be confirmed or excluded by needle core biopsy. Wide local excision of this tumor with 1 cm clear margins is associated with better overall survival compared to invasive breast cancer. We report the world’s oldest case of bilateral synchronous benign phyllodes tumor, which was managed with bilateral wide local excision with no recurrence after five years of follow-up.
